# Biomedical semantic indexing by deep neural network with multi-task learning

**DOI:** 10.1186/s12859-018-2534-2

**Published:** 2018-12-21

**Authors:** Yongping Du, Yunpeng Pan, Chencheng Wang, Junzhong Ji

**Affiliations:** Faculty of Information Technology, Beijing University of Technology, Beijing, China

**Keywords:** Multi-label classification, Biomedical semantic indexing, Data mining, Natural language processing, Multi-task learning, Word embedding

## Abstract

**Background:**

Biomedical semantic indexing is important for information retrieval and many other research fields in bioinformatics. It annotates biomedical citations with Medical Subject Headings. In face of unbalanced category distribution in the training data, sampling methods are difficult to apply for semantic indexing task.

**Results:**

In this paper, we present a novel deep serial multi-task learning model. The primary task treats the biomedical semantic indexing as a multi-label text classification issue that considers the relations of the labels. The auxiliary task is a regression task that predicts the MeSH number of the citation and provides hints for the network to make it converge faster. The experimental results on the BioASQ-Task5A open dataset show that our model outperforms the state-of-the-art solution “MTI”, proposed by the US National Library of Medicine. Further, it not only achieves the highest precision among all the solutions in BioASQ-Task5A but also has faster convergence speed compared with some naive deep learning methods.

**Conclusions:**

Rather than parallel in an ordinary multi-task structure, the tasks in our model are serial and tightly coupled. It can achieve satisfied performance without any handcrafted feature.

## Background

In order to index citations in MEDLINE, a life science and biomedicine journal database, National Library of Medicine (NLM) developed Medical Subject Headings (MeSH). Citations indexed by MeSH have been applied in fields such as query expansion [1], MEDLINE document clustering [2], enhancing search strategies for physical therapy [3] and so on. There are 28,472 MeSH main headings by 2017 [4]. Currently, the indexing work is performed by a group of qualified NLM staff, given the full text of each citation in MEDLINE. The task is becoming more and more tough on account of the annually increasing number of citations in MEDLINE (869,666 in 2016, and has approximately 8% increase over 2015 [5]). This fact leads to the manual semantic indexing task to be very inefficient and financially expensive. For example, the average cost of indexing each citation was reported to be around $9.4 [6].

The main challenge of the BioASQ task can be concluded as follows.

### Insufficient information

The participants are only provided with the name of the journal where the citations were published, the titles and the abstracts of the citations due to the limit of authority. By contrast, MeSH indexing experts of NLM have the full articles. Apparently, there are lots of useful information in the full article, which is not available to the participants.

### The large amount of MeSH

There are 28,472 MeSH by 2017. On the contrary, the average number of MeSH in each citation is 13 [6], thus there are much more negative labels than positive labels for each citation which increase the difficulty of indexing.

### Unbalanced distribution of MeSH in the MEDLINE

According to the research of Ke Liu et al. [6], the most frequent MeSH “Human” appears in 8,152,852 citations, while the 25,000th frequent MeSH “Pandanaceae” appears only in 31 citations in total 12,504,999 MEDLINE citations. As a result, there are not enough positive samples to learn the correct assignment of the infrequent MeSH.

Many research works have addressed the problem of biomedical semantic indexing by a wide variety of methods, and the most recent powerful methods have mainly used machine learning methods.

For example, the “Medical Text Indexers” (MTI) [7] of NLM annotated biomedical citations with Unified Medical Language System (UMLS) [8] using MetaMap [9]. It used Restrict-to-MeSH approach and the k-Nearest Neighbor (k-NN) algorithm. MTI is one of the most advanced method for indexing biomedical citations. It is also the baseline solution of BioASQ challenge task A [10], an international competition for automatically annotating new MEDLINE citations with MeSH. Liu et al. [6] proposed model “MeSHLabeler” which extracted several different features: the result of a MeSH classifier, the scores from the nearest neighbor citations, the MeSH and their synonyms directly found in the title or abstracts. “MeSHLabeler” integrated these features into a learning to rank framework [11]. It outperformed the MTI of the day and got the best performance in 2014 BioASQ challenge Task A.

Yuqing Mao and Zhiyong Lu [12] proposed “MeSH Now” which obtains an initial list of MeSH candidates from similar documents found by k-NN. A learning to rank algorithm is used to rank these MeSH candidates, and some hand-crafted rules are used for post-processing and top-ranked MeSH selection.

The “MetaLabeler” proposed by Tsoumakas [13] addressed the multi-label classification problem as *N* binary classification problems [14] and solved them using linear Support Vector Machine(SVM), where *N* is the number of MeSH. The MeSH were ranked in terms of the SVM prediction score of each classifier. A regression model which is independent of the classification models was trained to predict K, the number of MeSH for each citation, and used it to select the top K MeSH in the ranked list.

The methods mentioned above successfully integrated machine learning model with the knowledge resource and achieved encouraging results. However, they have two kinds of shortcomings. Firstly, they can’t represent the semantic of citations well by treating words as atomic symbols that ignore the words relation. Secondly, the machine learning methods currently used, such as SVM, k-NN and learning to rank model, need feature engineering in which researchers have to choose the features that fed to the machine learning model. The tedious feature engineering task not only requires domain knowledge but also lacks flexibility because some of the machine learning models lack interpretability in feature selection.

In this paper, we propose a deep learning model [15] with a serial multi-task learning structure to address the deficiency of the common methods for large-scale biomedical semantic indexing. We represent the citations as a sequence of word2vec [16] vectors. A bidirectional Gated Recurrent Unit (BGRU) [17] is used to take words order into consideration and generate the hidden representation of the citations. Finally, we design a serial multi-task structure [18] to get the model’s output, which contains a primary multi-label classification task and a serial auxiliary regression task. The model outperforms the state-of-art MTI in F-measure and precision, and it achieves the highest precision among all the solutions in BioASQ Task 5A. Furthermore, the experiments show that the deep neural network with a serial multi-task paradigm converges significantly faster.

We also try an interesting experiment in which the semantic indexing task is seen as generating labels given the representation of a citation. We use Wasserstein Generative Adversarial Nets(WGAN) to address the label generating issue.

## Methods

**Fig. 1:**
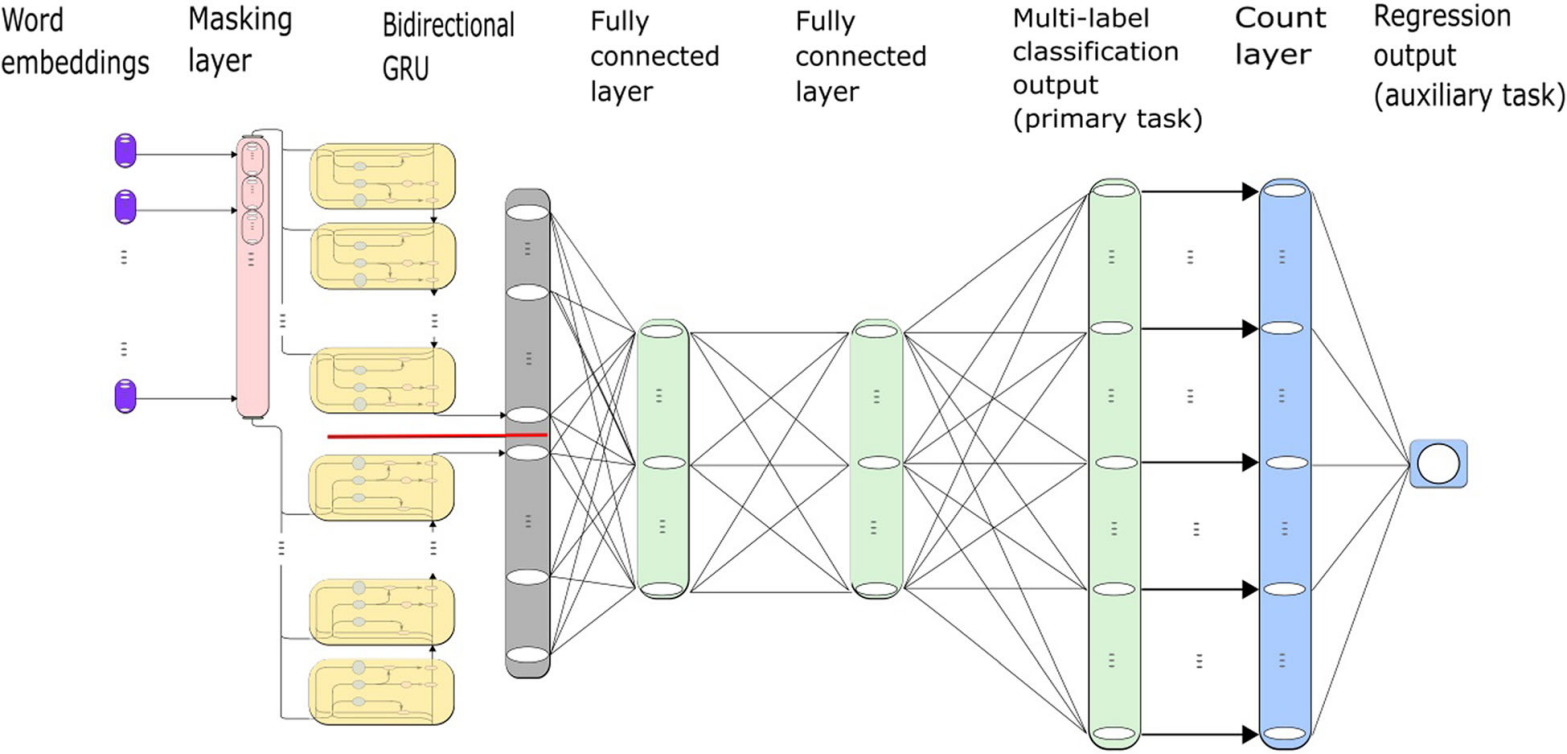
Deep Serial Multi-Task Learning Model (SMTL). The purple part is the sequence of word embedding (**seq**) that represents the training citation. The pink part is the masking layer for unifying the length of each **seq**. The yellow part denotes the BGRU and the two directions of which are separated by a red line. The gray part is the output of the BGRU, which is the hidden state. The green part includes three fully connected layers and the rightmost one is the output layer of the primary classification. The blue part represents the structure of the SMTL, of which the rightmost one is the output layer of the auxiliary regression

We proposed a deep serial multi-task learning model (SMTL) to solve biomedical semantic indexing problems. The outline of it is illustrated in Fig. 1.

We map the words in each citations to word2vec vectors that were pre-trained on 10,876,004 English abstracts in PubMed [19]. We use word embedding in consideration of the fact that it has overwhelming advantages [20] over other count based word representation methods.

We truncate or pad the input sequences to 360 words and feed the sequences to Bidirectional Recurrent Neural Network (BRNN) [21] to get the hidden representation. Gated Recurrent Units (GRU) [22] is used as the RNN cell. We stack three fully connected layers on top of the bidirectional GRU to perform the classification task. In order to alleviate the impact of the unbalanced data and to let multiple relevant tasks inform each other, we design an auxiliary regression task which adds up the elements of the output vector by the primary multi-label classification task. In addition, we use backpropagation algorithm to optimize the regression loss and the batch normalization [23] are adopted to speed up the training process.

### Neural semantic word embedding

Word2vec word embedding can be seen as a predictive-based language model for the word and the context, which embedded the semantic information of the words. The most famous example of word2vec embedding is “vector(“king”) – vector(“man”) + vector(“woman”) ≈ vector(“queen”)” [24]. Baroni et al. [20] showed that this kind of neural semantic word embedding is superior to count-based distributional semantic models and other kinds of semantic representation in most of the Natural Language Processing tasks.Word2vec has two kinds of models and they are the Continuous Bag-of-Words model (CBOW) and the Skip-Gram model [16].

**Fig. 2:**
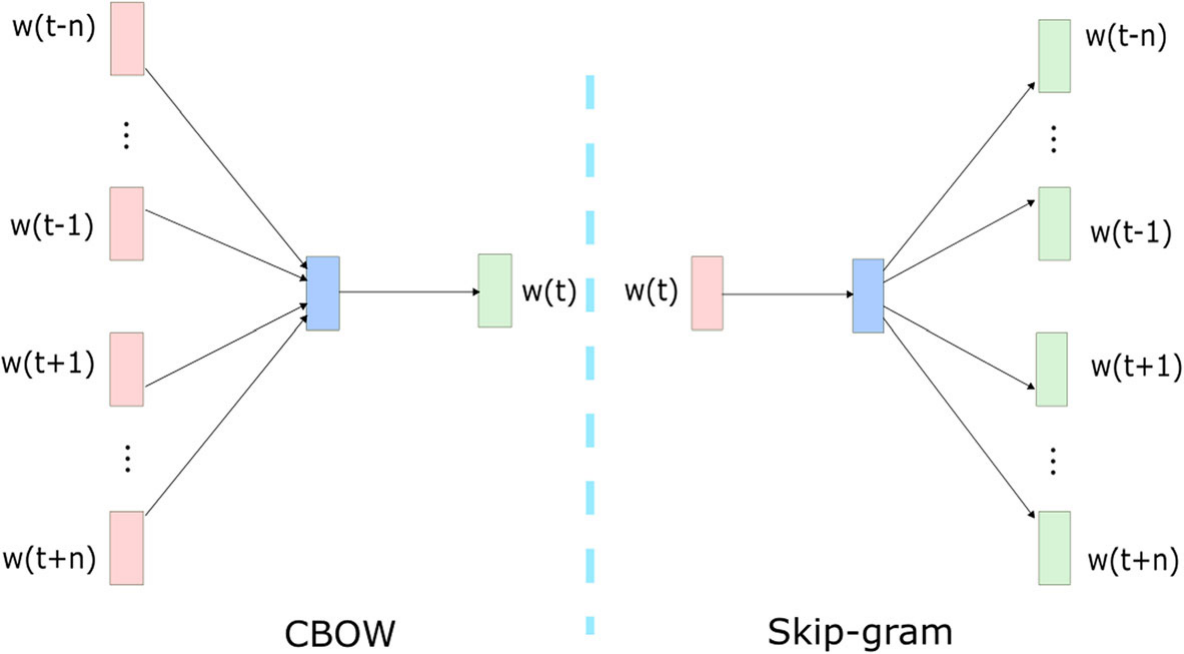
The CBOW Model and the Skip-gram Model. The CBOW Model uses the previous n words w(t-1)…w(t-n) and the subsequent n words w(t + 1)…w(t + n) to predict the target word w(t). The Skip-gram Model uses the target word w(t) to predict the surrounding words w(t-n)…w(t-1), w(t + 1)…w(t + n)

The CBOW model makes use of both the previous and subsequent *n*words around the target word to predict the target word *w*_*t*_. Conversely, the Skip-Gram model uses the center word to predict the surrounding words. They are shown in Fig. 2.

The objective function of CBOW is represented as Eq. 1.

1 $$ {J}_{\theta }=\frac{1}{\mathrm{T}}\sum \limits_{t=1}^T\log p\left({w}_t|{w}_{t-n},\cdots, {w}_{t-1},{w}_{t+1},\cdots, {w}_{t+n}\right) $$

The objective function of Skip-Gram model is represented as Eq. 2.

2 $$ {J}_{\theta }=\frac{1}{T}\sum \limits_{t=1}^T\sum \limits_{j=-n}^n\log p\left({w}_{t+j}|{w}_t\right)\kern1em ,\left(j\ne 0\right) $$

In order to get the word embedding, it is needed to maximize the objective function by maximizing the conditional probability. After the process of maximization, the network parameters corresponding to the words are the expected word embedding. We can simply implement the objective function using softmax function as illustrated in Eqs. 3 and 4, where *w*_*O*_denotes the surrounding words, *w*_*I*_denotes the center word, **v**represent the input embedding, **v**^'^represents the output embedding and *V*represents the size of the vocabulary.

3 $$ p\left({w}_I|{w}_O\right)=\frac{\exp \left({\mathbf{v}}_{w_t}^{\hbox{'}\mathrm{T}}{\mathbf{v}}_{w_I}\right)}{\sum_{w_i\in V}\exp \left({\mathbf{v}}_{w_i}^{\hbox{'}\mathrm{T}}{\mathbf{v}}_{w_O}\right)} $$

4 $$ p\left({w}_O|{w}_I\right)=\frac{\exp \left({\mathbf{v}}_{w_O}^{\hbox{'}\mathrm{T}}{\mathbf{v}}_{w_I}\right)}{\sum_{w=1}^V\exp \left({\mathbf{v}}_w^{\hbox{'}\mathrm{T}}{\mathbf{v}}_{w_I}\right)} $$

However, softmax function is computationally complex since the denominator involves all the words in the vocabulary which could be huge in practice, thus word2vec actually uses other more efficient methods instead of softmax function to represent the objective function.

In this paper, we use the pre-trained word embedding of 1,701,632 words provided by BioASQ which are trained on 10,876,004 English abstracts of biomedical articles from PubMed using skip-gram algorithm.

### Bidirectional gated recurrent unit

**Fig. 3:**
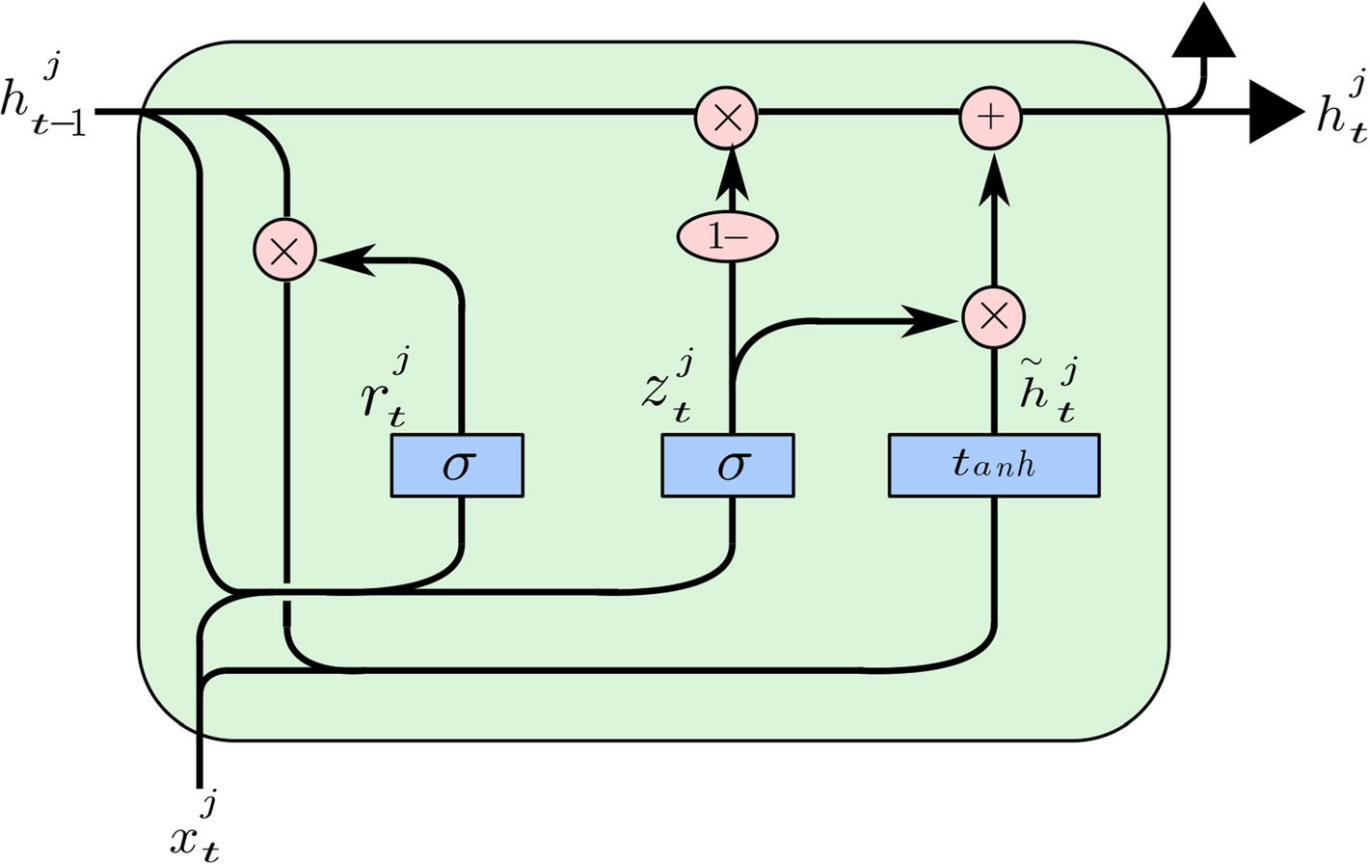
The unit of GRU. ${z}_t^j$is an update gate, where the superscript*j*indicates the update gate is the *j*thelement in the update gate vector**z**_*t*_, the subscript *t*indicates the time step *t*. ${r}_t^j$denotes a reset gate. ${h}_{t-1}^j$ represents a hidden state of the time step *t* ‐ 1, ${h}_t^j$ represents a hidden state of the time step t and ${\overset{\sim }{h}}_t^j$is a candidate activation

Gated recurrent unit (GRU) is designed to resist gradient vanishing and exploding problems of the Recurrent Neural Network (RNN) and it has the ability to learn to forget or update the recurrent hidden state according to the context. The unit of GRU is illustrated in Fig. 3.

It can be seen that the GRU unit has an update gate and a reset gate. The update gate ${z}_t^j$decides how much the unit update its content where *t*represents the time step *t* and *j* denotes the *j*thelement of the update gate vector **z**_*t*_. The reset gate ${r}_t^j$decides how much the new candidate value ${\overset{\sim }{h}}_t^j$consider the previous hidden state ${h}_{t\hbox{-} 1}^j$.

The update gate ${z}_t^j$ is computed by Eq. 5.

5 $$ {z}_t^j=\sigma {\left({\mathbf{W}}_z{\mathbf{x}}_t+{\mathbf{U}}_z{\mathbf{h}}_{t-1}\right)}^j $$

where **W**_**Z**_ denotes the input weights matrix, **U**_**Z**_ denotes the recurrent weights matrix for the update gate, **x**_**t**_ is the input vector of the unit on the time step *t* and *σ*denotes the element-wise sigmoid function.

Similarly, the reset gate ${r}_t^j$ is computed by Eq. 6.

6 $$ {r}_t^j=\sigma {\left({\mathbf{W}}_r{\mathbf{x}}_t+{\mathbf{U}}_r{\mathbf{h}}_{t-1}\right)}^j $$

where **W**_*r*_ denotes the input weights matrix, **U**_*r*_ denotes the recurrent weights matrix and **h**_*t* − 1_ is the hidden state of the previous time step.

The activation ${h}_t^j$of the GRU is a linear interpolation between candidate activation ${\overset{\sim }{h}}_t^j$and the previous activation ${h}_{t-1}^j$:

7 $$ {h}_t^j=\left(1-{z}_t^j\right){h}_{t-1}^j+{z}_t^j{\overset{\sim }{h}}_t^j $$

The candidate update activation ${\overset{\sim }{h}}_t^j$ is computed by Eq. 8.

8 $$ {\overset{\sim }{h}}_t^j=\tanh {\left({\mathbf{Wx}}_t+\mathbf{U}\left({\mathbf{r}}_t\bullet {\mathbf{h}}_{t-1}\right)\right)}^j $$

where **W** is the input weights and **U** is the recurrent weight matrix, • means elementwise multiplication.

The structure of GRU has the ability to capture dependencies over different time scales. In the units that learn to capture short-term dependencies, the reset gates tend to be active frequently. Meanwhile, in the units that capture long-term dependencies, the update gates tend to be more active. Most importantly, GRU can effectively alleviate the gradient vanishing and exploding problem which is the main disadvantage of standard RNNs. It makes the training process much easier.

**Fig. 4:**
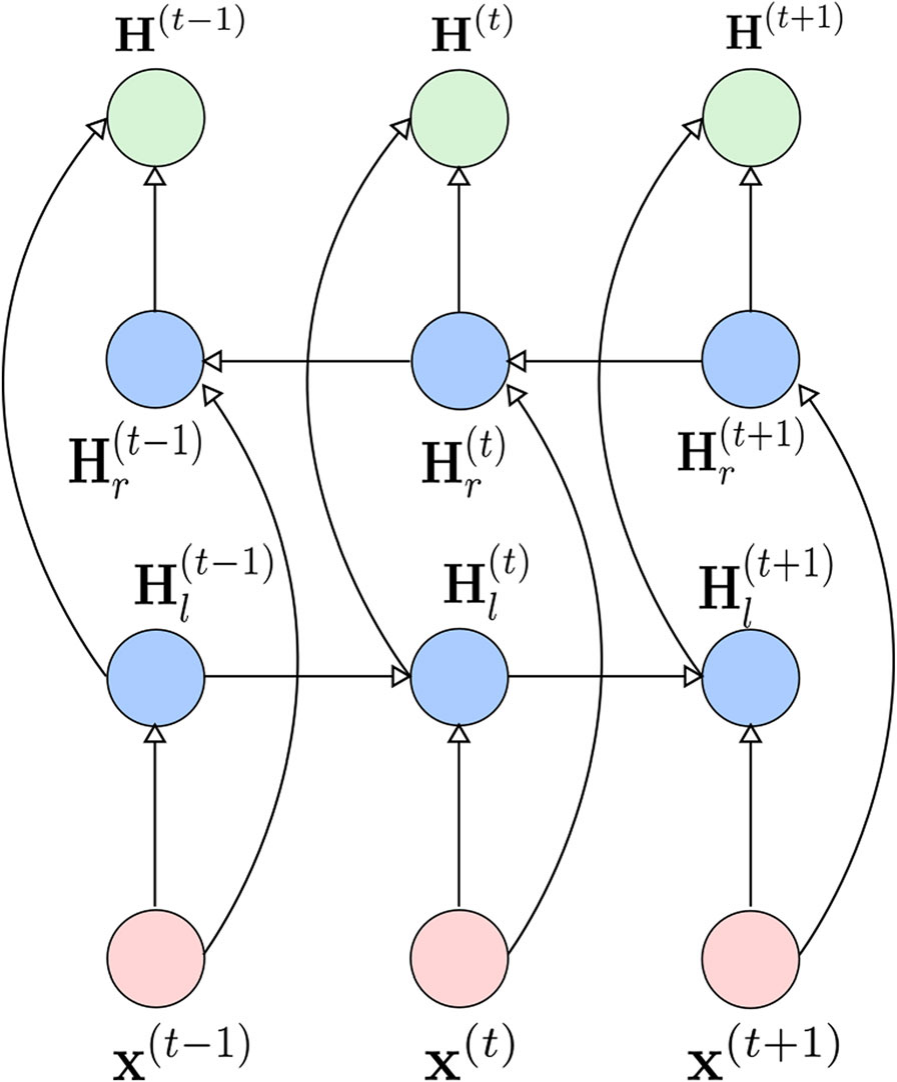
The Structure of BRNN. The pink circles annotated with **x** denotes the input vectors, the blue circles annotated with **H**_*l*_ and **H**_*r*_ denote the output of the recurrent hidden units in two different directions, and the green circles annotated with **H** denotes the output of the BRNN, which is the hidden state vector generated by concatenating the recurrent hidden states from two directions

Bidirectional recurrent neural network (BRNN) increases the amount of information available to the hidden representation of each time step allowing the recurrent units to use both the previous and the future information in the sequence. A bidirectional diagram is illustrated in Fig. 4.

Here, **H**_*l*_ denotes the output of the recurrent hidden units that propagate the information forward in time (from the left to the right) and **H**_*r*_ denotes the output of the recurrent hidden units that propagate the information backward in time(from the right to the left). Thus at each time step*t*, the output activations are computed by considering both the recurrent activation ${\mathbf{H}}_l^{\left(t-1\right)}$of the previous time step and the recurrent activation ${\mathbf{H}}_r^{\left(t+1\right)}$of the next time step. In this paper, we use GRU as the units of the BRNN, and concatenate **H**_*l*_ and **H**_*r*_ to get the hidden state **H**.

The bidirectional structure utilizes more information, thus strengthening the neural network’s ability of representation. Some researchers have claimed that they got better performance in the research fields of machine translation [25], speech recognition [26] and so on. We also find that Bidirectional GRU has better performance than plain unidirectional GRU in our model.

### Deep serial multi-task learning model

In multi-task learning (MTL) paradigm [18], more than one tasks are trained at the same time. The related tasks share part of the network structure, representation and the features extracted from each other so that these tasks can inform each other to learn better. Because the representation considers the need for all the tasks, the multi-task neural network tends to have a higher ability of generalization. Unrelated tasks can also benefit from the multi-task learning paradigm, too [27].

The loss of the MTL network is given by Eq. 9.

9 $$ {L}_{MTL}={L}_{pri}+\sum \limits_{k=1}^n{\lambda}_k{L}_k $$

where *L*_*pri*_ refers to the loss of the primary task and *L*_*k*_ refers to the loss of the *k*th auxiliary task, *λ*_*k*_ is the relative importance factor of the auxiliary task with respect to the primary task.

**Fig. 5:**
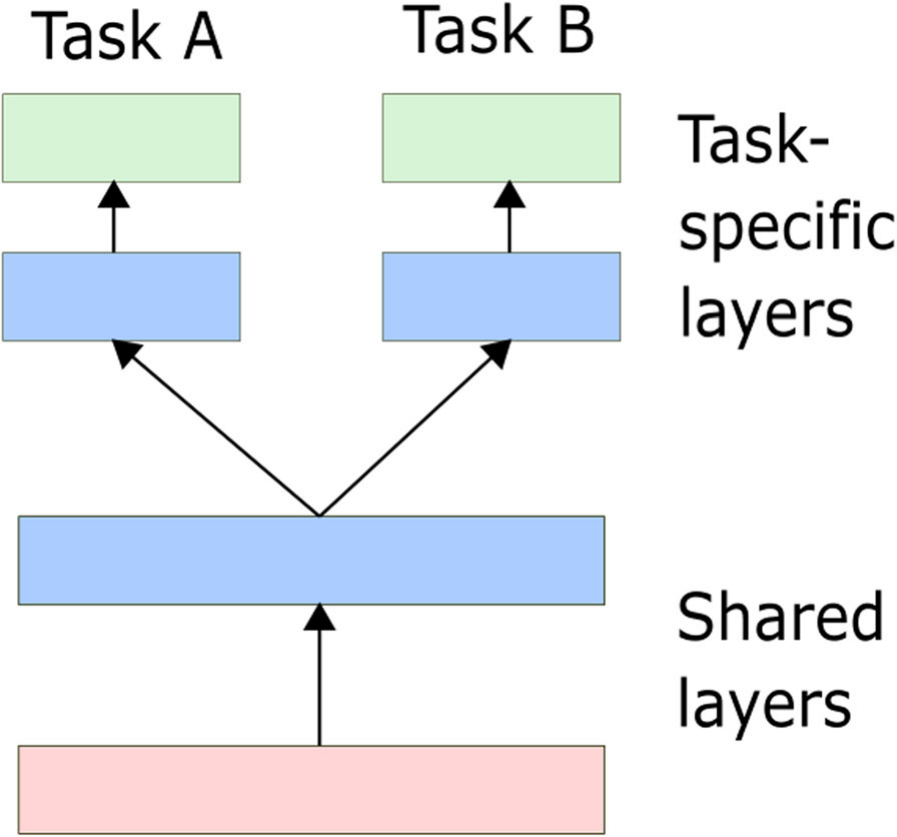
The Ordinary Hard Parameter Sharing MTL Structure. Task A and B share the stationary hidden layers and keep some task-specific output layers

MTL has two main paradigms and the first one is the hard parameter sharing MTL paradigm, which shares the hidden layers among all the tasks and keeps several task-specific output layers as illustrated in Fig. 5.

The other one is the soft parameter sharing MTL paradigm [28], in which each task has its own relatively independent model and there are extra structures and parameters among these models for learning to share information.

Neural network based multi-label classification is naturally a multi-task learning process, in which the classification of each label can be seen as one task which shares the representation with the other classification tasks for other labels. The multi-label classification process in the neural network considers the relation between different labels, thus it has better performance over traditional machine learning methods that treat the multi-label classification as independent binary classifications.

As for the model we propose in this paper, we combine two related tasks in the novel serial multi-label learning structure. The primary task is the multi-label classification task, in which each label refers to a candidate MeSH. When a label is assigned the value of 1, it means that the corresponding MeSH should be assigned to the citation.

**Fig. 6:**
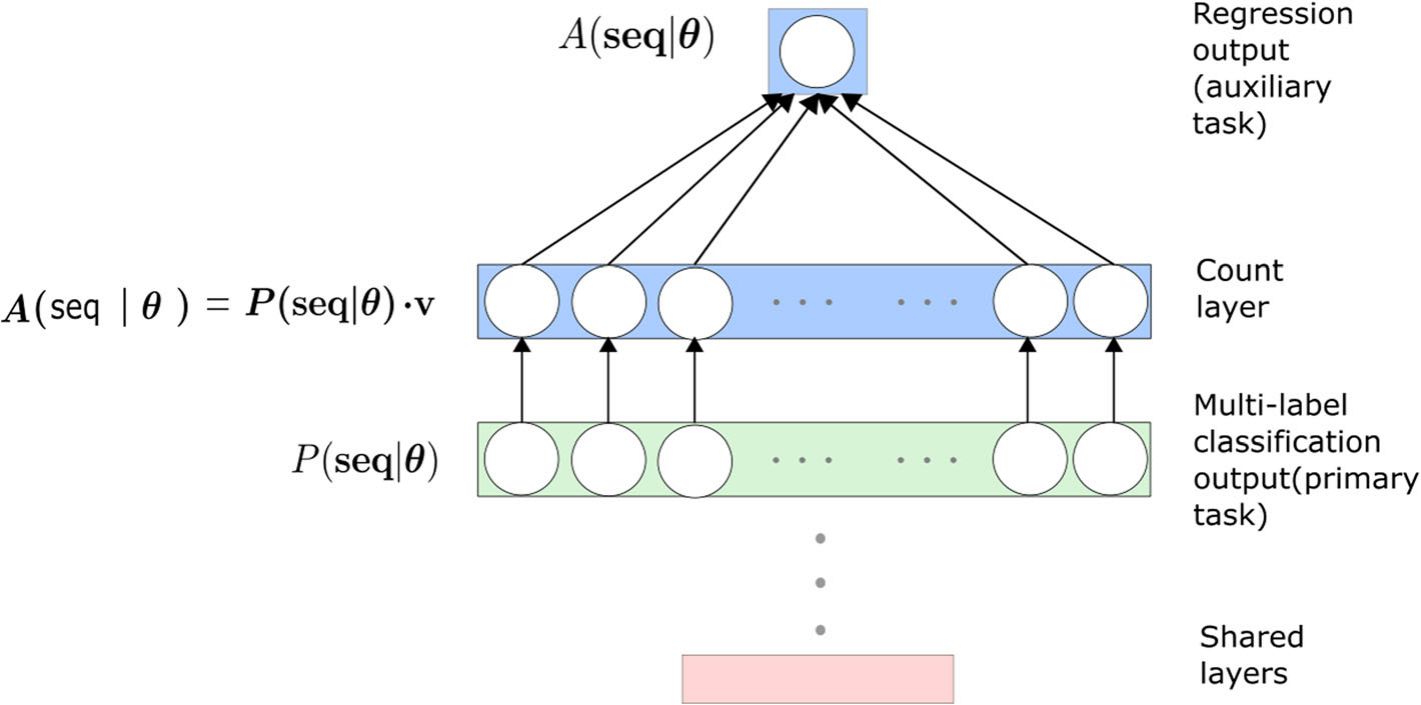
The structure of the multi-task learning paradigm. The primary multi-label classification task is denoted as *P*(**seq**| **θ**), the auxiliary regression task is denoted as *A*(**seq**| **θ**)and the relationship between *P*and *A*is described in eq. 10

The structure of the serial multi-task paradigm is shown in Fig. 6. The count layer implements the operation described in Eq. 10. The primary classification task can be formalized as *P*(**seq**| **θ**), where **θ** denotes the weights of the neural network and **seq** denotes the sequence of word embedding which is the input of the model.

The auxiliary task is a regression task and we formalize it as *A*(**seq**| **θ**). The calculation of the auxiliary task is shown in Eq. 10.

10 $$ R=A\left(\mathbf{seq}|\boldsymbol{\uptheta} \right)=P\left(\mathbf{seq}|\boldsymbol{\uptheta} \right)\cdot \mathbf{v} $$

where *R* is a scalar which denotes the output of the auxiliary regression task, and it is the prediction for the total number of the labels (MeSH) that the citation has. Here, **v** is the vector (1, 1, ⋯, 1) with 28,472 dimensions.

The operation demonstrated by Eq. 10 is equal to the process that counts the total number of the labels predicted by the primary classification task *P*(**seq**| **θ**)and uses it as the output of the auxiliary regression task *A*(**seq**| **θ**).

During the optimization, the weights of the network will be updated to ensure the low loss of both the primary and auxiliary task. Thus this serial multi-task structure can deal with the primary task by knowing the total number of labels for the corresponding citations. The auxiliary task can inform the primary task and the generalization ability of the network is higher. We name the structure as the Serial Multi-Task Learning model (SMTL) for multi-label classification.

We use cross-entropy to measure the loss of the primary classification network, mean square error is used to measure the loss of the auxiliary regression network. Therefore, the loss function of SMTL is:

11 $$ L={L}_{pri}+{L}_{aux} $$

Here, *L*_*pri*_is the cross-entropy loss of the primary classification network and *L*_*aux*_is the mean square loss of the auxiliary regression network.

12 $$ {L}_{pri}=-\frac{1}{N}\sum \limits_{i=1}^N\sum \limits_{j=1}^{28472}\left(\begin{array}{l}{\mathbf{y}}_j^i\log P{\left({\mathbf{seq}}^i|\boldsymbol{\uptheta} \right)}_j\\ {}+\left(1-{\mathbf{y}}_j^i\right)\log \left(1-P{\left({\mathbf{seq}}^i|\boldsymbol{\uptheta} \right)}_j\right)\end{array}\right) $$

13 $$ {L}_{aux}=\frac{\lambda }{N}\sum \limits_{i=1}^N{\left({\mathbf{z}}^i-A\left({\mathbf{seq}}^i|\boldsymbol{\uptheta} \right)\right)}^2 $$

Here, **y**represents the ground true label of the primary classification task and **z** represents the label of the regression task, **seq**denotes the input of the model which is a word embedding sequence representing the training citation. The superscript *i* denotes the *i*thtraining sample of a selected mini-batch, and the subscript indicates the element in the corresponding vector.

The SMTL model makes use of the hard parameter sharing MTL paradigm, but it differs from the ordinary hard parameter sharing MTL model. The ordinary one’s tasks are parallel while the tasks in our model are serial and the auxiliary task is based on the primary task. It informs the primary task more directly and makes the network learn faster. SMTL shows high performance in the experiment.

The algorithm of SMTL is described in Algorithm 1.

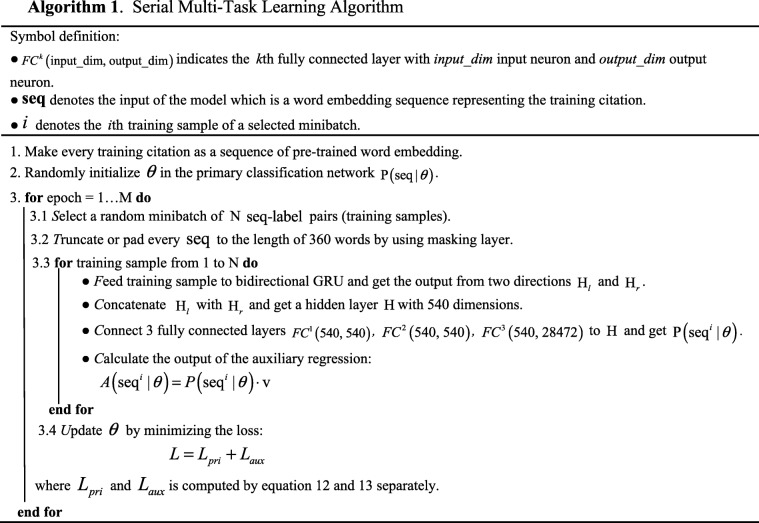



Wasserstein Generative Adversarial Networks (WGAN) [29] is a promising generative model in which a generative model G captures the data distribution, and a discriminative model D estimates the probability that a sample came from the training data rather than G. We try to address the semantic indexing issue via Wasserstein Generative Adversarial Nets (WGAN). The hidden state of the recurrent network in the trained SMTL model is a representation of the input citation, and we use it as the input of the generator to achieve a label combination. A discriminator is trained to distinguish the generated label combinations and the real label combinations. In the inference stage, we use the trained generator to predict the label combinations of the citations.

## Results and discussion

### Data set and details of the experiment

We use BioASQ Task 5A dataset [10] as training set, and the entire dataset contains 12,504,999 labeled citations annotated by NLM staff.

The 2017 BioASQ challenge Task A released the test data in many batches, and the participants’ submitted solutions were evaluated by the performance on the batches. In order to compare our solution with other participants’ approaches sufficiently, we choose the last batch which is “week 5 batch 3” as the test data since more teams joined this evaluation than any other test batches.

With regard to the detail of the experiment, we use 200 dimensions word2vec embedding to represent the word. We use the masking layers to generate citations with variable length. The batch normalization layers are used to make the network less fragile during the training process and it also alleviates the overfitting problem. For the initialization of the GRU, we initialize the weights for the input vectors with Xavier uniform initializer [30]. The bias is initialized with zero vector. The hidden state of GRU is a vector of 270 dimensions, so that after the concatenation of the two directions, the hidden state is a vector of 540 dimensions. The two fully-connected layers are both of 540 dimensions. For the optimization process, we use Adam optimizer [31]. The deep learning library Theano [32] and Keras [33] are used to build our model. We use the first 3 million out of the total 12 million data in the Bioasq dataset as the training data and the model is trained on a Nvidia 1080ti GPU.

### Experimental results

We design several other models and compare them with SMTL in the experiment.

**SMTL**: Our final model, which utilize a serial multi-task learning structure and bidirectional GRU.

**Model A**: An ordinary hard parameter sharing MTL model with Bidirectional GRU, and the MTL structure is demonstrated in Fig. 5 where Task A denotes the multi-label classification and Task B denotes the regression task.

**Model B**: A multi-label classification model with Bidirectional GRU.

**Model C**: A SMTL model which has a unidirectional GRU instead of a bidirectional GRU.

**Fig. 7:**
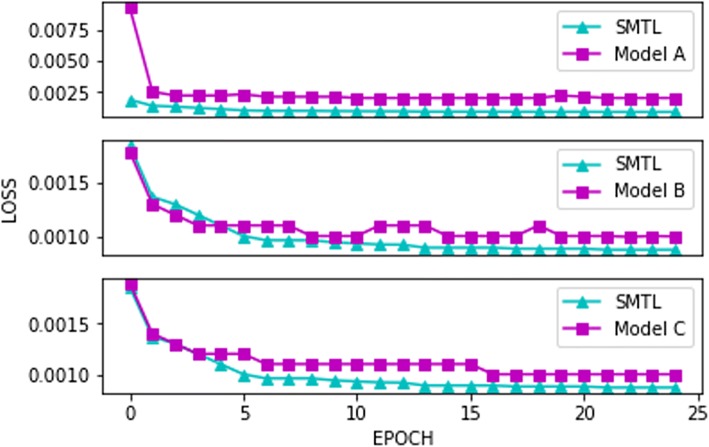
The classification loss during the training process for different Model. The result shows that network with SMTL converges faster and has better performance than networks with an ordinary parallel multi-task learning structure (Model A) and network without a multi-task structure (Model B). It also shows that BGRU has better performance than unidirectional GRU (Model C) in SMTL

The binary cross-entropy loss of the multi-label classification task in different models during the training process is demonstrated in Fig. 7.

The main evaluation metrics in the BioASQ challenge Task A are Precision, Recall, and F-measure.

**Fig. 8:**
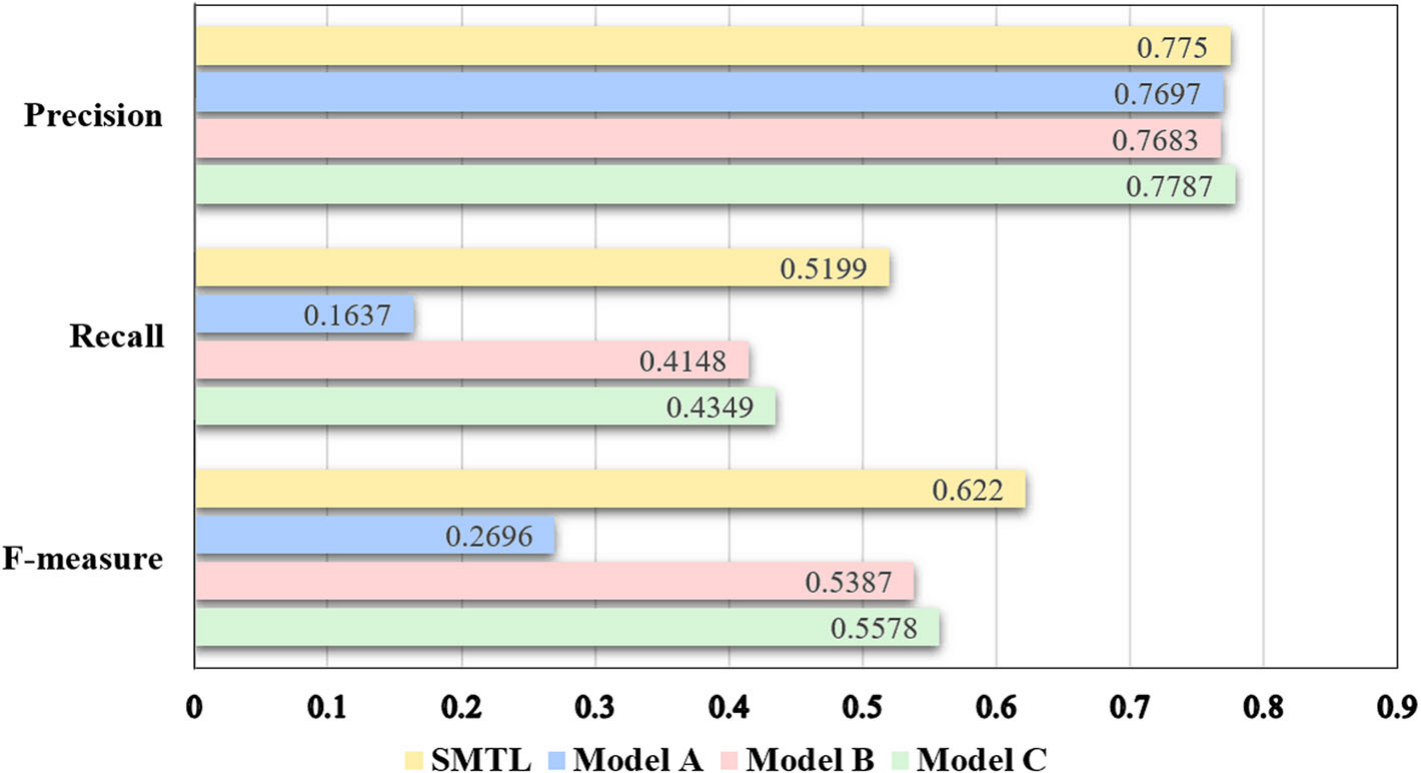
Performance comparison of different models. SMTL performs better than Model A, B and C. The tightly coupled serial structure informs the tasks more so that the model spends less time learning the relationship between the primary and auxiliary tasks

The performance evaluation results are demonstrated in Fig. 8.

**Fig. 9:**
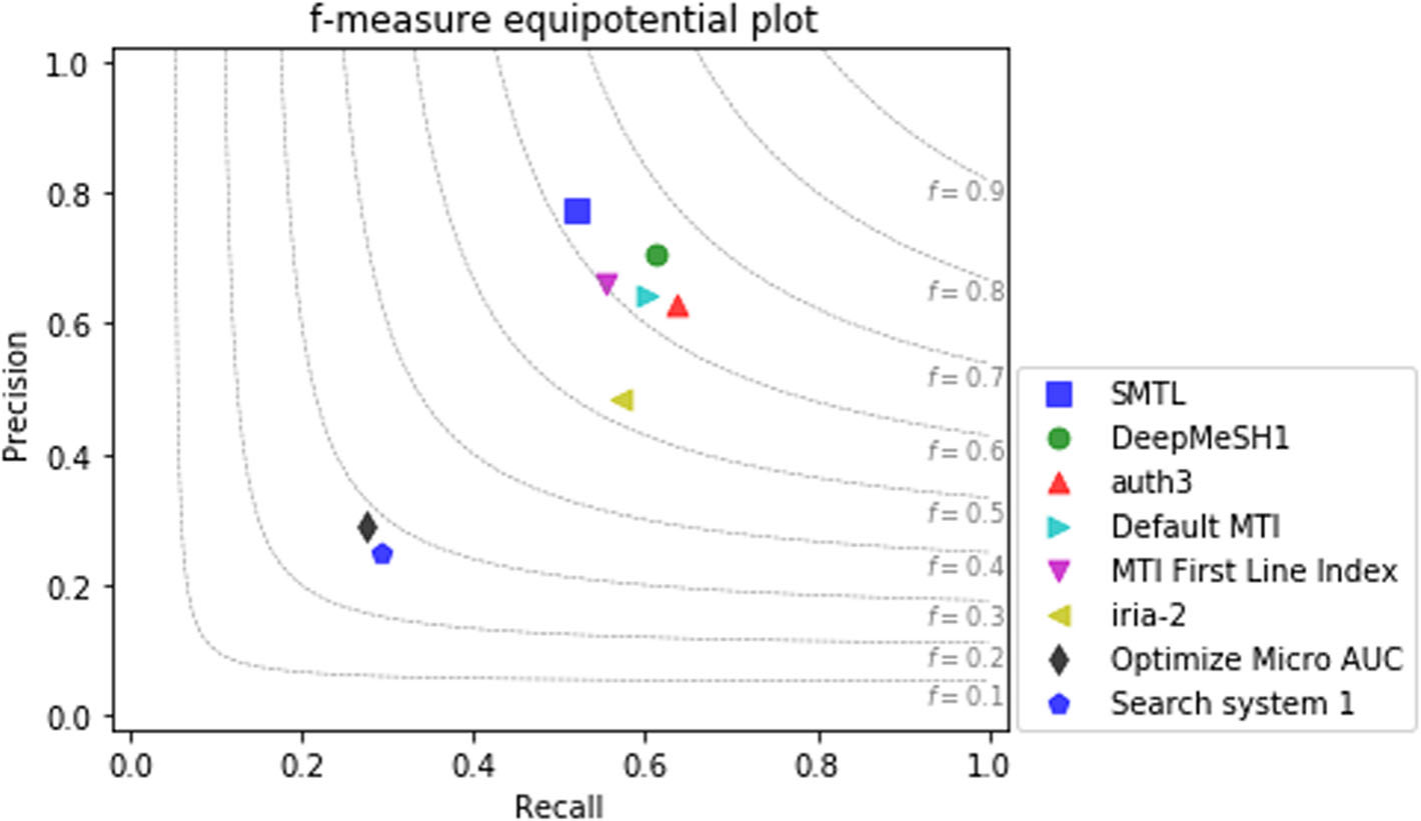
F-measure equipotential plot of different solutions in 2017 BioASQ Task 5A. The best solutions from each team are exhibited in the Fig. 9. The plot shows that without any hand-craft feature or any sophisticated rule design, SMTL (the blue rectangle) achieves the highest precision among solutions of all the participants in 2017 BioASQ Task 5A

The performance comparison with other solutions submitted by different participant teams in 2017 [34] is shown in Table 1 and Fig. 9. It’s worth noticing that the solution “DeepMeSH1” [35] is the champion in the 2017 BioASQ Task 5A competition. Our experimental result labelled as “SMTL” achieves the highest precision among all the other solutions.

**Table 1:** Performance comparison with different solutions in 2017 BioASQ task 5A

System	Precision	Recall	F-measure
SMTL	0.7750	0.5199	0.6220
DeepMeSH1	0.7052	0.6135	0.6561
auth3	0.6277	0.6366	0.6321
Default MTI	0.6408	0.6021	0.6209
MTI First Line Index	0.6624	0.5534	0.6030
iria-2	0.4853	0.5721	0.5251
Optimize Micro AUC	0.2890	0.2739	0.2812
Search system 1	0.2488	0.2907	0.2681

As demonstrated in Table 1 and Fig. 9, our SMTL outperforms the state-of-art solution MTI proposed by NLM on both precision and F-measure. As a reference, only the solutions from two participant teams [35, 36] beat MTI on F-measure in the 2017 BioASQ Task 5A challenge and neither of them adopted deep learning methods. Bidirectional GRU is good at capturing long term dependency in sequence data and does not need explicit feature extraction. SMTL structure increases the generalization ability of the model and makes the model converge faster in practice.

We use mean square error(MSE) as the metric for the auxiliary regression task, and the MSE loss on the test set is 27.54.

As depicted in Fig. 1, the recurrent neural network gives a representation of the input word sequences(citations). We use it as the input of the fully connected layers. And then the classification issue can also be viewed as training a generative model to generate a vector of 28,472 dimensions as the labels assigned to the citations.

We also try to use a WGAN framework to resolve the semantic indexing problem. The presentations in the trained SMTL are used as the prior vectors of G in WGAN to generate the 28,472 dimension label vectors. And 3 layers of fully connected neural networks are designed as a discriminative network to evaluate the authenticity of the generated vector.

The performance of the WGAN framework with the recurrent presentation is illustrated in Table 2 along with SMTL.

**Table 2:** Performance comparison between WGAN framework and SMTL

System	Precision	Recall	F-measure
WGAN Framework	0.6376	0.5486	0.5898
SMTL	0.7750	0.5199	0.6220

Since the model G and D in our WGAN framework are just fully connected neural networks with only 3 layers, it still remains great potential of the WGAN with the recurrent presentation.

## Conclusions

We propose a novel deep serial multi-task learning model to address the issue of biomedical semantic indexing. The traditional methods ignore the relations among labels and need complicated feature engineering. Our model uses the word2vec word embedding to represent the words in the citations, and the Bidirectional GRU is used to create the representation of the data. This multi-task learning structure is different from an ordinary one because the auxiliary task originates directly from the primary task and the two tasks compose a serial structure. The regression task is motivated by dynamic threshold for classification task on unbalanced data. Without any handcrafted feature, our model outperforms the state-of-art baseline solution MTI in F-measure, and it has higher precision than the best solution “DeepMeSH1” in 2017 BioASQ Task 5A.

Furthermore, we are going to explore more auxiliary tasks to inform the multi-label classification task and apply the attention mechanism to our model for higher performance. And we will also pay more attention to investigate the possibilities of using WGAN for semantic indexing task.

## Abbreviations

BGRU: Bidirectional Gated Recurrent Unit
BRNN: Bidirectional Recurrent Neural Network
CBOW: Continuous Bag-of-Words
GRU: Gated Recurrent Units
K-NN: K-Nearest Neighbor
MeSH: Medical Subject Headings
MTI: Medical Text Indexers
MTL: Multi-Task Learning
NLM: National Library of Medicine
RNN: Recurrent Neural Network
SMTL: Serial Multi-task Learning model
SVM: Support Vector Machine
UMLS: Unified Medical Language System
WGAN: Wasserstein Generative Adversarial Networks
